# Visfatin Mediates SCLC Cells Migration across Brain Endothelial Cells through Upregulation of CCL2

**DOI:** 10.3390/ijms160511439

**Published:** 2015-05-18

**Authors:** Tingting Liu, Ziwei Miao, Jiusheng Jiang, Shuai Yuan, Wengang Fang, Bo Li, Yuhua Chen

**Affiliations:** Key Lab of Cell Biology, Ministry of Public Health, Key Lab of Medical Cell Biology, Ministry of Education, Department of Developmental Biology, China Medical University, No. 77 Puhe Road, Shenyang North New Area, Shenyang 110122, China; E-Mails: liutingting19881123@hotmail.com (T.L.); zwmiao@mail.cmu.edu.cn (Z.M.); jjs19890504@hotmail.com (J.J.); yuanshuai43210@126.com (S.Y.); fangwengang@126.com (W.F.); bli@mail.cmu.edu.cn (B.L.)

**Keywords:** brain metastasis, SCLC, visfatin, transendothelial migration, blood–brain barrier (BBB), CC chemokine ligand 2 (CCL2)

## Abstract

Small-cell lung cancer (SCLC) is characterized as an aggressive tumor with brain metastasis. Although preventing SCLC metastasis to the brain is immensely important for survival, the molecular mechanisms of SCLC cells penetrating the blood–brain barrier (BBB) are largely unknown. Recently, visfatin has been considered as a novel pro-inflammatory adipocytokine involved in various cancers. Herein, we present evidence that elevated levels of visfatin in the serum of SCLC patients were associated with brain metastasis, and visfain was increased in NCI-H446 cells, a SCLC cell line, during interacting with human brain microvascular endothelial cells (HBMEC). Using *in vitro* BBB model, we found that visfatin could promote NCI-H446 cells migration across HBMEC monolayer, while the effect was inhibited by knockdown of visfatin. Furthermore, our findings indicated that CC chemokine ligand 2 (CCL2) was involved in visfatin-mediated NCI-H446 cells transendothelial migtation. Results also showed that the upregulation of CCL2 in the co-culture system was reversed by blockade of visfatin. In particular, visfatin-induced CCL2 was attenuated by specific inhibitor of PI3K/Akt signaling in NCI-H446 cells. Taken together, we demonstrated that visfatin was a prospective target for SCLC metastasis to brain, and understanding the molecular mediators would lead to effective strategies for inhibition of SCLC brain metastasis.

## 1. Introduction

Small-cell lung cancer (SCLC) is aggressive and rapidly metastasizing to the brain, with poor prognosis [1]. A vital event of brain metastasis (BM) is that tumor cells migrate through the blood–brain barrier (BBB), which is distinct from other metastasis sites [2]. The BBB, which is mainly constituted of brain microvascular endothelial cells, maintains brain homeostasis [3]. In case of tumors burden, tight junctions (TJs) between the brain endothelial cells become stretched out, leading to an increase in the permeability of the BBB [4]. Our previous studies had indicated that SCLC cells disrupted the integrity of TJs between human brain microvascular endothelial cells (HBMEC), resulting in SCLC cells transendothelial migration [5,6]. These data suggested that breakdown of the BBB is an important step for tumor cells metastasis to brain. Therefore, novel therapeutic strategies for SCLC BM are urgently needed.

Visfatin, also identified as nicotinamide phosphoribosyltransferase (NAMPT) and pre-B-cell colony-enhancing factor (PBEF), is predominantly released by visceral fat tissue, particularly by the macrophages [7,8]. It has been reported that visfatin contributes to energy production through regulation of NAD^+^ and regulates intracellular ATP levels in mammalian cells [9]. In addition, elevated plasma visfatin concentrations have been reported in the early stages of diabetic nephropathy [10]. Visfatin can also be considered as a new pro-inflammatory adipocytokine, which is involved in various malignant tumors, including colon, stomach, pancreas, liver, prostate, and breast cancers [11,12]. However, the involvement of visfatin in the pathogenesis of SCLC remains undetermined.

In the current study, we have identified the role of visfatin in SCLC cells migration across brain endothelial cells. That is, visfatin could induce the upregulation of CCL2 in SCLC cells, and the elevated CCL2 “open” the TJs and promoted SCLC cells migration across brain endothelial cells.

## 2. Results

### 2.1. Visfatin Contributed to NCI-H446 Cells Migration across HBMEC Monolayer

**Figure 1:**
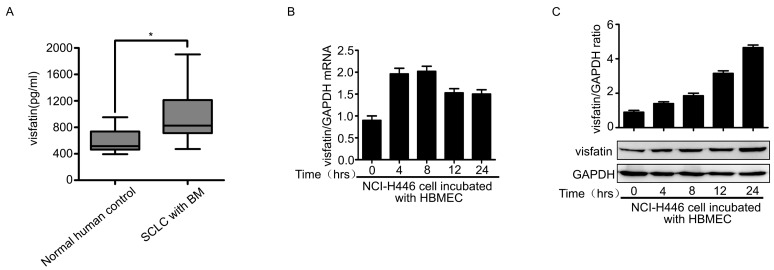

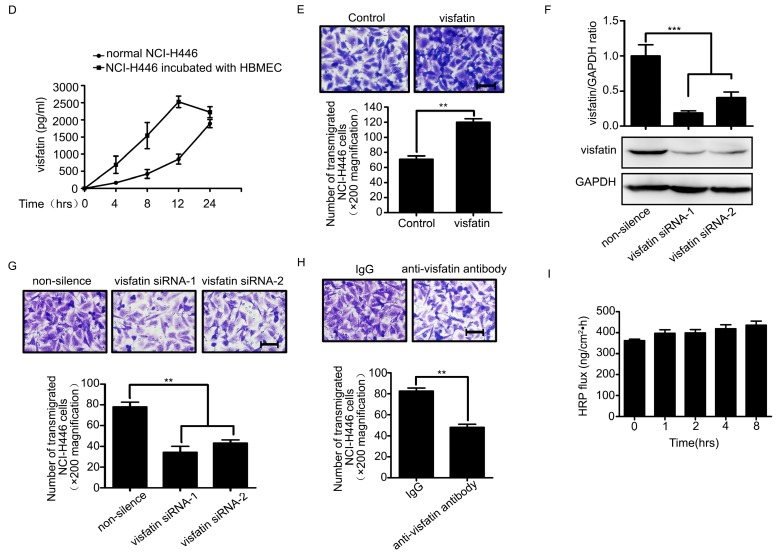
(**A**) ELISA analysis for visfatin in the serum of normal human control (*n* = 21) and SCLC patients with BM (*n* = 21); (**B**) mRNA levels of visfatin in NCI-H446 cells were analyzed during interacting with HBMEC by real-time PCR, with GAPDH as control; (**C**) protein levels of visfatin in NCI-H446 cells were analyzed during interacting with HBMEC by Western blot, with GAPDH as control; (**D**) the levels of visfatin in the supernatant were measured by ELISA during co-culture of NCI-H446 cells and HBMEC; (**E**) transendothelial migration of NCI-H446 cells in the presence of recombinant human visfatin protein (100 ng/mL). Scale bar: 50 μm; (**F**) NCI-H446 cells were transiently transfected with visfatin siRNA, with non-silencing siRNA as control. After 48 h, the expression of visfatin was analyzed by Western blot, with GAPDH as control; (**G**) knockdown of visfatin in NCI-H446 cells was subjected to transendothelial migration assay. Scale bar: 50 μm; (**H**) transendothelial migration of NCI-H446 cells was analyzed in the presence of anti-visfatin antibody (10 μg/mL). Scale bar: 50 μm; (**I**) the HRP flux through HBMEC monolayer was assessed by treating with visfatin (100 ng/mL) for indicated times. Values are means ± SD of three independent experiments done in triplicate. * *p* < 0.05; ** *p* < 0.01; *** *p* < 0.001.

It had been reported that increased expression of visfatin was closely associated with the pathogenesis tumor. Therefore, we examined the visfatin in the serum samples of SCLC patients with BM. The results showed that the levels of visfatin in SCLC patients with BM were significantly higher than that of normal specimens (Figure 1A). We further investigated whether the expression of visfatin in NCI-H446 cells was modulated during interacting with HBMEC. The experiments were performed according to the method as following: HBMEC were grown on alumina of the membrane inserts and NCI-H446 cells were grown on insert’s luminal side. After co-culture for indicated times, NCI-H446 cells were collected and the expression of visfatin was determined. As shown in Figure 1B,C, visfatin was significantly increased at mRNA and protein levels. Similarly, the results from ELISA assay also showed that the interaction of NCI-H446 cells and HBMEC induced the upregulation of visfatin in the co-culture cell supernatant (Figure 1D). These results suggested that visfatin might be associated with SCLC metastasis to brain.

Because tumor cells transendothelial migration was a key event in cancer metastasis, we evaluated the effect of visfatin on transendothelial migration of NCI-H446 cells using the *in vitro* BBB model [13,14]. As shown in Figure 1E, treatment with visfatin led to a significant increase in the tansendothelial migration of NCI-H446 cells as compared to control. To further characterize the involvement of visfatin in the process, specific siRNA targeting visfatin was used to knock down the expression of visfatin in NCI-H446 cells (Figure 1F). Subsequent results showed that the downregulation of visfatin significantly inhibited NCI-H446 cells transendothelial migration (Figure 1G). The experiment of antibody blockage showed the similar results (Figure 1H). It had been reported previously that SCLC cells disrupted the TJs between HBMEC, contributing to SCLC cells transendothelial migration [5,6]. To ascertain whether visfatin could impair the integrity of TJs between HBMEC, the paracellular permeability of HBMEC monolayer was assessed using the HRP flux assay. The results demonstrated that there were little change in the paracellular permeability of HBMEC monolayer after treatment with visfatin for the indicated times (Figure 1I). Taken together, these results suggested that visfatin might modulate several inflammatory factors, which were associated with NCI-H446 cells transendothelial migration.

### 2.2. CCL2 Was Involved in Visfatin-Mediated NCI-H446 Cells Transendothelial Migration

**Figure 2:**
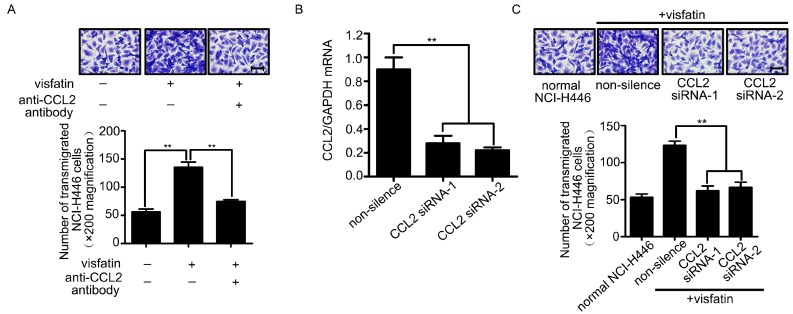
(**A**) The HBMEC monolayer was treated with visfatin followed by CCL2 neutralizing antibody (4 μg/mL), and then the migration of NCI-H446 cells through the HBMEC was assessed. Scale bar: 50 μm; (**B**) the efficiency of CCL2 siRNA in NCI-H446 cells was evaluated by real-time PCR. GAPDH was used as control; (**C**) Knockdown of CCL2 in NCI-H446 cells in the presence of visfatin was subjected to transendothelial migration assay. Values are means ± SD of three independent experiments done in triplicate. Scale bar: 50 μm. ** *p* < 0.01.

Recently, evidences showed that CCL2 was associated with breast tumor metastasis to brain [15]. Moreover, it was reported that visfatin was a positive regulator of CCL2 in human adipocytes [16]. To investigate whether CCL2 was involved in visfatin-mediated NCI-H446 cells transendothelial migration, a neutralizing antibody against CCL2 was used. The results showed that visfatin-mediated NCI-H446 cells transendothelial migration was suppressed by CCL2 neutralizing antibody (Figure 2A). Similarly, CCL2 silencing was verified by real-time PCR and the migration was also inhibited by knockdown of CCL2 in NCI-H446 cells (Figure 2B,C). These results suggested that visfatin-mediated NCI-H446 cells migration across HBMEC was dependent on CCL2. 

### 2.3. The Upregulation of CCL2 Was Induced by Visfatin in the Co-Culture System of NCI-H446 Cells and HBMEC

**Figure 3:**
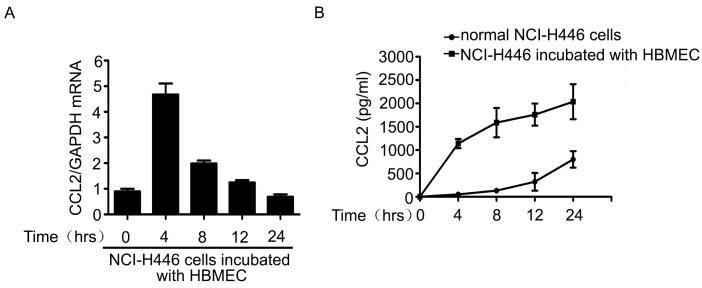

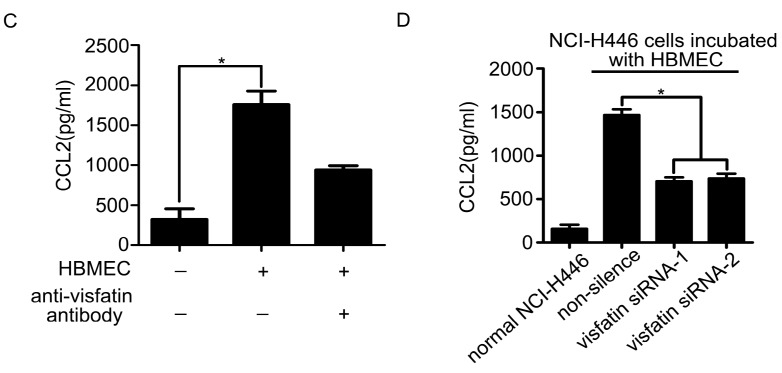
(**A**) mRNA levels of CCL2 in NCI-H446 cells were analyzed during interacting with HBMEC by real-time PCR, with GAPDH as control; (**B**) the levels of CCL2 in the supernatant were measured by ELISA during co-culture of NCI-H446 cells and HBMEC; (**C**) NCI-H446 cells were treated with HBMEC in the presence of anti-visfatin antibody, and then the expression of CCL2 was assessed by ELISA; (**D**) NCI-H446 cells were transfected with visfatin siRNA followed by incubating with HBMEC, and then the expression of CCL2 was assessed by ELISA. Values are means ± SD of three independent experiments done in triplicate. * *p* < 0.05.

The above results showed that CCL2 was also a mediator in the transendothelial migration of NCI-H446 cells. Therefore, the levels of CCL2 in the co-culture system of NCI-H446 cells and HBMEC were detected by real-time PCR and ELISA assay. As shown in Figure 3A, mRNA levels of CCL2 in NCI-H446 cells were significantly increased at 4 h. In addition, the release of CCL2 was significantly elevated in a time-dependent manner (Figure 3B). Our further investigation demonstrated that visfatin-neutralizing antibody led to a reduction of CCL2 in the co-culture cell supernatant (Figure 3C). Similarly, knockdown of visfatin in NCI-H446 cells also significantly attenuated the release of CCL2 (Figure 3D). These results suggested that visfatin upregulated the expression of CCL2 in the co-culture system of NCI-H446 cells and HBMEC.

### 2.4. PI3K/Akt Signaling Was Involved in Visfatin-Induced the Upregulation of CCL2

**Figure 4:**
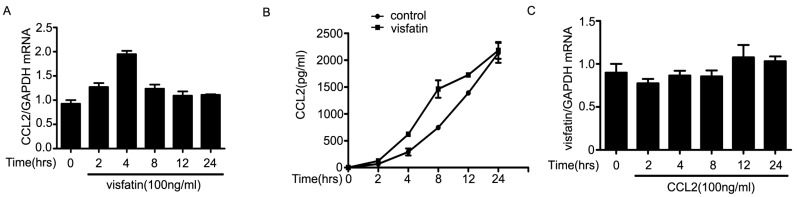

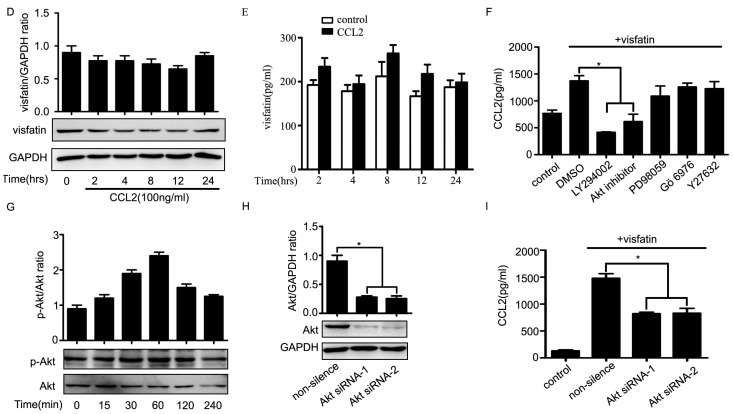
(**A**) mRNA levels of CCL2 were analyzed in visfatin (100 ng/mL)-treated NCI-H446 cells by real-time PCR, with GAPDH as control; (**B**) the levels of CCL2 derived from NCI-H446 cells were assessed in the presence of visfatin (100 ng/mL) by ELISA; (**C**) mRNA levels of visfatin derived from NCI-H446 cells were analyzed in the presence of CCL2 (100 ng/mL) by real-time PCR, with GAPDH as control; (**D**) protein levels of visfatin in NCI-H446 cells were measured in the presence of CCL2 (100 ng/mL) by Western blot, with GAPDH as control; (**E**) the levels of visfatin derived from NCI-H446 cells were measured in the presence of CCL2 (100 ng/mL) by ELISA; (**F**) NCI-H446 cells were pretreated with indicated inhibitors for 1 h followed by visfatin (100 ng/mL) for 8 h, and then the secretion of CCL2 was measured by ELISA; (**G**) visfatin for indicated times, and then the phosphorylation of Akt in NCI-H446 cells was examined by Western blot; (**H**) NCI-H446 cells were transiently transfected with Akt siRNA, with non-silencing siRNA as control. After 48 h, the expression of visfatin was analyzed by Western blot, with GAPDH as control; (**I**) knockdown of Akt in the NCI-H446 cells was subjected to ELISA assay to evaluate the production of CCL2. Values are means ± SD of three independent experiments done in triplicate. * *p* < 0.05.

Next, we sought to elucidate the molecular mechanisms of the regulation of visfatin on the expression of CCL2. The results showed that mRNA levels of CCL2 in NCI-H446 cells were increased and peaked at 4 h after treatment with visfatin (Figure 4A). Moreover, visfatin induced NCI-H446 cells to secrete CCL2 in a time dependent manner, and the peak was at 8 h (Figure 4B). However, the levels of visfatin in NCI-H446 cells were unchangeable after treatment with CCL2 (Figure 4C–E). To ascertain which signaling molecules might mediate the effect of visfatin-induced CCL2, several specific inhibitors, including LY294002, Akt inhibitor, PD98059, Gö6976, Y27632, were used for inhibition experiments. As shown in Figure 4F, LY294002 and Akt inhibitor notably diminished the secretion of CCL2 induced by visfatin, whereas PD98059, Gö6976 and Y27632 had no effects. Meanwhile, visfatin treatment also resulted in the phosphorylation of Akt (Figure 4G). To further confirm the above results, NCI-H446 cells were transfected with Akt siRNA to inhibit the expression of Akt (Figure 4H). The subsequent results showed that knockdown of Akt prevented visfatin-induced CCL2 release (Figure 4I). These results suggested that visfatin upregulated CCL2 in NCI-H446 cells via PI3K/Akt signaling.

## 3. Discussion

BM is a significant cause of mortality in patients. The majority of BM originates from lung cancer, breast cancer and melanoma [17,18,19]. During metastasis to brain, malignant tumor cells enter into the circulatory system through the endothelium (intravasation) and then attach to microvessel endothelial cells to invade the BBB (extravasation) [20]. In SCLC, 10% of patients have BM at time of primary tumor diagnosis [21]. However, the molecular basis for SCLC metastasis to brain is largely undetermined. In this study, we firstly clarified that autocrinal visfatin upregulated the expression of CCL2 in SCLC cells, which contributed to SCLC cells migration through brain endothelial cells.

Visfatin had also been identified as a target of anti-cancer therapy. Elevated visfatin expression was associated with malignant cancer behavior as well as adverse prognosis [22]. It was reported that visfatin was involved in lung carcinogenesis and the measurements of serum visfatin were proposed for diagnosis in non-small cell lung cancer (NSCLC) [23]. Shunsuke Okumura *et al.* also determined that attenuation of visfatin function suppressed growth of NSCLC through reduction of intracellular ATP levels [24]. Paradoxically, Antonio F. Santidrian *et* *al.* reported that knockdown of visfatin, the enzyme catalyzing the rate-limiting step of the NAD^+^ salvage pathway, enhanced metastatic aggressiveness in human breast cancer cells [25]. However, virtually no information was available regarding the role of visfatin in SCLC BM. Here, we showed that visfatin was elevated in the SCLC patients with BM, which suggested visfatin might be a potential biomarker for the brain metastastic state at the initial diagnosis of SCLC patients. Using *in vitro* BBB model, we found that SCLC cells derived-visfatin was enhanced during incubating with HBMEC. Importantly, visfatin promoted SCLC cells transendothelial migration. It was known that the first obstacle for metastatic tumor cells reaching the central nervous system (CNS) was the invasion of the BBB, and our previous study showed that SCLC cells could disrupt TJs and transmigrate through the BBB [5,6]. These studies suggested that visfatin might impact the integrity of the BBB to promote SCLC cells transendothelial migration. However, our data revealed that visfatin had little effect on the permeability of HBMEC monolayer. Therefore, the exact mechanisms of visfatin-mediated SCLC cells migration through brain endothelium remained elusive.

Accumulating evidences demonstrated that visfatin was identified as a proinflammatory adipocytokine and secreted extracellularly to upregulate inflammatory cytokines, such as TNF-α, IL-1β, IL-6 and CCL2 in rheumatoid arthritis synovial fibroblasts and monocytes in response to inflammatory stimuli [26,27]. These data provided a possibility that visfatin might serve as an autocrine regulation of inﬂammatory responses and might exert a permissive role for the activity of other chemokines, which were associated with SCLC cells transendothelial migration. Recent report pointed out that CCL2 was one of the most highly and transiently expressed chemokines during inflammation and implicated in the pathogenesis of cancer metastasis [28,29]. Moreover, existing reports in the literature demonstrated that CCL2 had a significant role in promoting prostate cancer cell extravasation into the bone microenvironment and also contributing to breast tumor metastasis to brain [15,30]. Meanwhile, CCL2 breached the BBB through downregulating the proteins of TJ complex, such as claudin5, ZO-1 and ZO-2 [31,32]. The above-mentioned data prompted us to hypothesize that CCL2 might implicated in visfatin-mediated SCLC cells transendothelial migration. In the present study, we confirmed that the blockade of CCL2 inhibited the ability of visfatin-induced SCLC cells transendothelial migration. Interestingly, we also found that SCLC cells-derived CCL2 was increased during incubating with HBMEC, while the upregulation of CCL2 was reversed by the blockade of visfatin. Accordingly, we validated that visfatin acted as a swift to modulate CCL2-induced disruption of the BBB, which contributed to SCLC cells transendothelial migration. Furthermore, we found that visfatin upregulated CCL2 in SCLC cells, whereas the levels of visfatin were unchangeable after treatment with CCL2. Taken together, SCLC cells-derived visfatin might enhance the levels of CCL2 in an autocrine manner, and the increased CCL2 disrupted the BBB to promote SCLC cells metastasis to brain.

To date, several signaling pathways, including JNK, PI3K, ERK, had been reported to contribute to visfatin-induced downstream CCL2 in various cell types [33,34]. To ascertain these cascades involved in the process of SCLC cells, several specific inhibitors were applied in the inhibition experiments. Only PI3K and Akt inhibitors effectively inhibited visfatin-induced CCL2 expression. We evaluated the activated status of Akt in SCLC cells after stimulation with visfatin. The finding indicated that treatment of visfatin resulted in phosphoylation of Akt. Subsequently, knockdown of Akt significantly attenuated the visfatin-mediated CCL2 secretion. Our results confirmed that visfatin enhanced CCL2 expression via PI3K/Akt-dependent mechanism.

In conclusion, this study provided a clear scenario for visfatin-mediated SCLC cells migration across the BBB. Initially, visfatin derived from SCLC cells active PI3K/Akt signaling, and then, upregulated the expression of CCL2 in an autocrine manner. Subsequently, the elevated CCL2 facilitated SCLC cells “open” the TJs followed by penetrating into the BBB. Our findings elucidated a novel target, visfatin, to prevent penetration of SCLC cells through the BBB and metastasis to brain.

## 4. Experimental Section

### 4.1. Patients and Specimens

The collection of serum samples were from 21 SCLC patients with BM who attended the Shengjing Hospital of China Medical University (Shenyang, China) from 2007 to 2010. A total of 21 serum samples of normal human were from Physical Examination Center in Shengjing Hospital of China Medical University (Shengyang, China). Institutional Review Board approval (16 March 2013) was obtained to procure and analyze all the samples used in this study.

### 4.2. Cell Lines and Cell Culture

Human brain microvascular endothelial cells were a generous gift from Kwang Sik Kim (Johns Hopkins University, Baltimore, MD, USA). They were cultured in RPMI 1640 medium supplemented with 10% fetal bovine serum (Gibco, Carlsbad, CA, USA) and 10% Nu-serum (BD Biosciences, Franklin Lakes, NJ, USA). The human small cell lung cancer cell line, NCI-H446, was obtained from American Type Culture Collection (ATCC, Rockville, MD, USA), and maintained in RPMI 1640 medium containing 10% fetal bovine serum.

### 4.3. Real-Time PCR

The total RNA isolated with TRIzol reagent (Invitrogen, Carlsbad, CA, USA) was reverse transcribed using M-MLV reverse transcriptase (Promega, Madison, WI, USA). Real-time PCR was performed on Applied BioSystems 7500 system with a SYBR^®^ Select Master Mix kit (Invitrogen, Carlsbad, CA, USA) according to the manufacturer’s protocol. The primer sequences for human visfatin were 5'-AGCAGCAGAACACAGTACCA-3' and 5'-GCTGACCACAGATACAGGCA-3'. The primer sequences for human CCL2 were 5'-TCAAACTGAAGCTCGCACTCT-3' and 5'-CATTGATTGCATCTGGCTGAG-3'. The primer sequences for human GAPDH were 5'-GAAGGTGAAGGTCGGAGTC-3' and 5'-GAAGATGGTGATGGGATTTC-3'. The comparative cycle threshold (*C*_t_) method was used to calculate the relative gene expression. GAPDH was used as internal control.

### 4.4. Cell Fractionation and Western Blot

Cells at 80% confluence were lysed by radioimmunoprecipitation assay (RIPA) buffer (50 mM Tris–HCl, 150 mM NaCl, 1% NP-40, 0.5% sodium deoxycholate, 0.1% sodium dodecyl sulfate) containing protease inhibitor cocktail (Roche Diagnostics, Indianapolis, IN, USA). The samples were separated by SDS-PAGE and then transferred to polyvinylidene difluoride (PVDF) membrane (Millipore, Billerica, MA, USA). The PVDF membrane was blocked with 5% fat-free dry milk and incubated with anti-visfatin (1:1000, Santa Cruz, CA, USA), anti-Akt (1:1000, Santa Cruz, San Francisco, CA, USA), anti-phopsho-Akt (1:1000, Millipore, Billerica, MA, USA) and anti-GAPDH (1:5000, KangChen Bio-tech Inc, Shanghai, China) antibodies overnight at 4 °C. Blots were subsequently probed with horseradish peroxidase-conjugated anti-mouse secondary antibodies (Santa Cruz, San Francisco, CA, USA). Immunoreactive bands were visualized by Super Signal West Pico chemiluminescent substrate (Pierce, Rockford, IL, USA) on Fujifilm LAS-3000 Imager (FujiFilm, Tokyo, Japan). Relative quantitations of Western blot were done using Image J software (National Institutes of Health, Bethesda, MD, USA).

### 4.5. Enzyme-Linked Immunosorbent Assay

The visfatin and CCL2 in human serum were analyzed by commercially available enzyme-linked immunoassay (ELISA) kits (Uscn Life Science Inc., Wuhan, China) according to the manufacturer’s instructions.

### 4.6. Transendothelial Migration Assay

Ten thousand HBMEC were seeded on the upper chamber of transwell insert with 8 μm pore size (Corning Inc., Cambridge, MA, USA) in 24-well plates and cultured for 5 days to form confluent monolayer. One hundred thousand NCI-H446 cells were subsequently loaded into the top of insert. After incubation for 8 h, cells that migrated to the bottom surface were fixed in methanol and stained with crystal violet. Non-migrating cells were scraped gently with cotton wool from the upper surface of insert and migrated cells were photographed under a microscope.

### 4.7. siRNA Transfection

For visfatin, CCL2 and Akt knockdown, predesigned human siRNA was synthesized and purified by GenePharma (Shanghai, China). One hundred picomoles siRNA oligonucleotides were introduced into NCI-H446 cells by transfection using Lipofectamine 2000 reagent (Invitrogen, Carlsbad, CA, USA) according to the manufacturer’s instructions. Forty-eight hours after transfection, cells were used for experiments. Nonspecific oligonucleotides were used as control. The siRNA sequences of visfatin were No. 1 (UAGUCAUUCAAUCUGGUAUUG) and No. 2 (AGCGAUAGCUAUGACAUUUAU). The siRNA sequences of CCL2 were No. 1 (UCAUAGCAGCCACCUUCAUUC) and No. 2 (GAUGUGAAACAUUAUGCCUUA). The siRNA sequences of Akt were No. 1 (GUGCCAUGAUCUGUAUUUAdTdT) and No. 2 (GAGACUGACACCAGGUAUUdTdT).

### 4.8. HRP Flux Measurement

Ten thousand HBMEC were grown on the apical chamber of 24-well transwell plates with 0.4 μm pore size (Corning Inc., Corning, MA, USA) for 4 days. Immediately after the addition of visfatin (Axxora, Nottingham, UK), HRP (0.4 mg/mL; Sigma–Aldrich, St. Louis, MO, USA) was added to the apical chamber. After incubation for 1 h, the medium from the lower chamber were collected and the HRP flux through HBMEC monolayer was determined by spectrophotometric measurement. The HRP flux was expressed as nanogram passed per cm^2^ surface area per hour.

### 4.9. Statistical Analysis

Data were presented as means ± SD from three independent experiments. Statistical analysis was carried out using Student’s *t-*test and one-way ANOVA. *p* < 0.05 was considered as statistical significance.
